# Substrate‐Independent Magnetic Bistability in Monolayers of the Single‐Molecule Magnet Dy_2_ScN@C_80_ on Metals and Insulators

**DOI:** 10.1002/anie.201913955

**Published:** 2020-01-24

**Authors:** Denis S. Krylov, Sebastian Schimmel, Vasilii Dubrovin, Fupin Liu, T. T. Nhung Nguyen, Lukas Spree, Chia‐Hsiang Chen, Georgios Velkos, Claudiu Bulbucan, Rasmus Westerström, Michał Studniarek, Jan Dreiser, Christian Hess, Bernd Büchner, Stanislav M. Avdoshenko, Alexey A. Popov

**Affiliations:** ^1^ Leibniz Institute for Solid State and Materials Research Helmholtzstraße 20 01069 Dresden Germany; ^2^ Center for Quantum Nanoscience Institute for Basic Science (IBS) Seoul Republic of Korea; ^3^ Department of Medicinal and Applied Chemistry Kaohsiung Medical University Kaohsiung 807 Taiwan; ^4^ The division of synchrotron radiation research Lund University 22100 Lund Sweden; ^5^ Swiss Light Source Paul Scherrer Institute 5232 Villigen PSI Switzerland

**Keywords:** endohedral metallofullerenes, monolayers, scanning probe microscopy, single-molecule magnets, XMCD

## Abstract

Magnetic hysteresis is demonstrated for monolayers of the single‐molecule magnet (SMM) Dy_2_ScN@C_80_ deposited on Au(111), Ag(100), and MgO|Ag(100) surfaces by vacuum sublimation. The topography and electronic structure of Dy_2_ScN@C_80_ adsorbed on Au(111) were studied by STM. X‐ray magnetic CD studies show that the Dy_2_ScN@C_80_ monolayers exhibit similarly broad magnetic hysteresis independent on the substrate used, but the orientation of the Dy_2_ScN cluster depends strongly on the surface. DFT calculations show that the extent of the electronic interaction of the fullerene molecules with the surface is increasing dramatically from MgO to Au(111) and Ag(100). However, the charge redistribution at the fullerene‐surface interface is fully absorbed by the carbon cage, leaving the state of the endohedral cluster intact. This Faraday cage effect of the fullerene preserves the magnetic bistability of fullerene‐SMMs on conducting substrates and facilitates their application in molecular spintronics.

## Graphical abstract

**Monolayers of Dy_2_ScN@C_80_** exhibit magnetic hysteresis on Au, Ag, and MgO|Ag substrates. The substrates influence the ordering of the endohedral cluster, but do not affect the width of the hysteresis. The carbon cage protects the endohedral magnetic cluster and thus ensures that it sustains magnetic bistability.

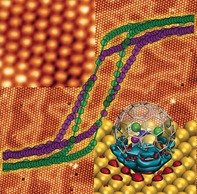

## Introduction

Single‐molecule magnets (SMMs) are molecular materials exhibiting magnetic bistability and slow relaxation of magnetization.^1^ Since these properties should be preserved even for single molecules, the prospects of SMMs in information storage and spintronics^2^ have been pushing the field toward developing new molecules with better SMM performance and higher operating temperatures.^3^ So far, the vast majority of the studies of SMMs have been performed for their powders and crystals. At the same time, realization of the true advantages of SMMs over bulk magnetic materials require the scaling down to 2D, 1D, and eventually a single‐molecule level. Investigation of the magnetic properties of monolayers is thus the next logical step after the bulk SMM behavior is established.^4^ However, formation of monolayers requires certain chemical and/or thermal stability, which many SMMs do not have, whereas surface techniques for the study of the sample morphology or magnetism are rather complicated. As a result, the SMMs, whose magnetic properties have been studied on surfaces, are very rare in comparison to hundreds of known SMM compounds. The scaling down to monolayer level also raises a question of the substrate influence on the SMM properties of adsorbed molecules. In particular, interaction with conducting electrons is believed to be deteriorating for the SMM behavior. So far, magnetic hysteresis on conducting substrates was demonstrated only for three types of SMMs: LnPc_2_ (Ln=Tb, Dy),^5^ Fe_4_,^6^ and metallofullerenes Dy_1(2)_Sc_2(1)_N@C_80_.^7^

Endohedral metallofullerenes^8^ form a special class of SMMs in which lanthanides are encapsulated within the carbon cage.^9^ This allows stabilization of unusual lanthanide clusters with strong single‐ion magnetic anisotropy^10^ or giant exchange coupling.^11^ Furthermore, high thermal stability of fullerenes enables formation of monolayers by sublimation.^12^ The first study of the surface magnetism of the fullerene‐SMM monolayer was performed by X‐ray magnetic circular dichroism (XMCD) technique for Dy_2_ScN@C_80_ on Rh(111) and indeed revealed a magnetic bistability.7a However, magnetic hysteresis of the monolayer was considerably narrower than for a multilayer sample. A broader hysteresis than on Rh(111) was observed for Dy_2_ScN@C_80_ on *h*‐BN|Rh(111).7b Here we demonstrate that the SMM properties of Dy_2_ScN@C_80_ monolayers on Au(111) and Ag(100), as well on a thin film of insulator MgO grown on Ag(100), do not depend on the substrate, which highlights the robustness of fullerene SMMs and the protective function of the carbon cage.

## Results and Discussion

**Figure 1:**
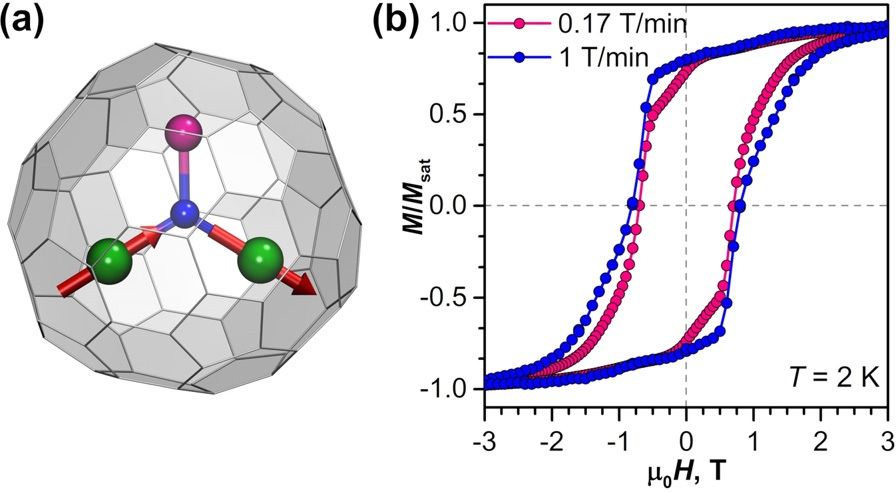
a) Molecular structure of Dy_2_ScN@C_80_ (Dy green, N blue, Sc magenta; magnetic moments of Dy ions are visualized as red arrows). b) Magnetic hysteresis measured for the powder sample of Dy_2_ScN@C_80_ by SQUID magnetometry at *T*=2 K with magnetic field sweep rates of 0.17 and 1 T min^−1^.

The molecule of Dy_2_ScN@C_80_ is built from an icosahedral carbon cage C_80_‐*I_h_*, which encapsulates a planar metal nitride cluster Dy_2_ScN (Figure 1 a). The nitride ion N^3−^ is located in the center of a triangle formed by metal ions in their formal oxidation state of 3+. Dy−N bond lengths in Dy_2_ScN@C_80_ are shorter than 2.1 Å,10b which results in a strong uniaxial ligand field imposed by the nitride onto Dy^3+^ ions.10b, 13 The magnetic ground state of each Dy^3+^ ion is a Kramers doublet with *m_J_*=±15/2 and a quantization axis aligned along the corresponding Dy−N bond. Very large ligand‐field splitting ensures that single‐ion magnetic moments are locked to the Dy−N bonds up to high temperature. The intramolecular interactions between magnetic moments of Dy ions are predominantly ferromagnetic (Figure 1 a), and the state with anti‐parallel alignment of magnetic moment is higher in energy by 0.8 meV (10 K).10b, 14 Dy_2_ScN@C_80_ is a single‐molecule magnet with a blocking temperature of magnetization near 8 K and a 100 s blocking temperature of 5 K.10b At 2 K, the powder sample of Dy_2_ScN@C_80_ shows magnetic hysteresis with a coercive field of 0.7–0.8 T (Figure 1 b).

To ensure the suitability of the evaporated thin films of Dy_2_ScN@C_80_ for the studies of magnetic properties by XMCD technique, deposition of the fullerene onto Au(111) substrate and characterization of the monolayer properties by scanning tunneling microscopy (STM) were first performed. The molecules were deposited under ultra‐high‐vacuum conditions (*p*≤10^−9^ mbar) via organic molecular beam epitaxy using a home build evaporator. Typical evaporation conditions were 20 minutes at 450–460 °C. The substrate was kept at room temperature during deposition, and STM studies were also performed at room temperature using Omicron VT‐STM/AFM microscope.

**Figure 2:**
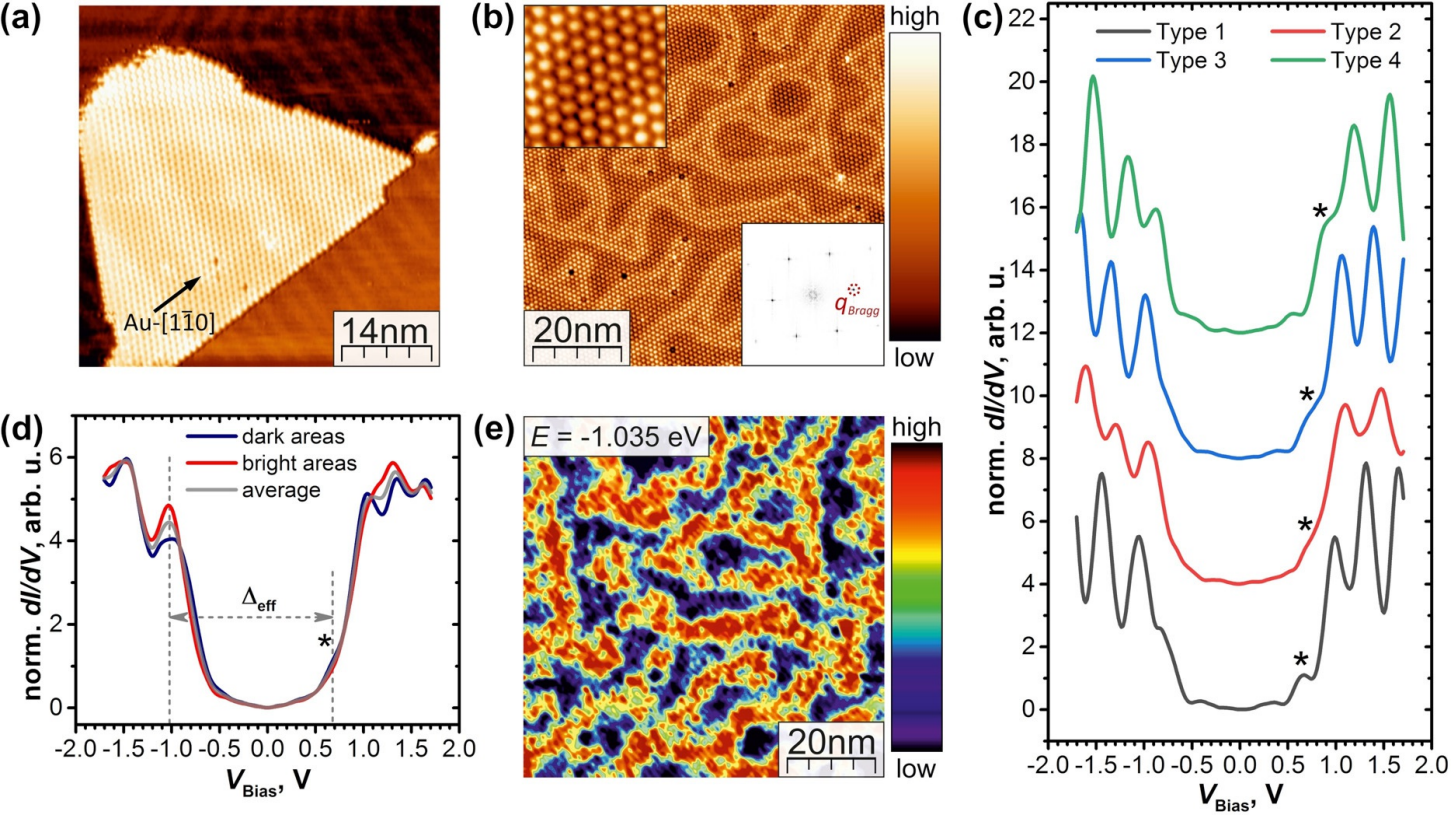
a) Constant current topography image of an hcp monolayer island of Dy_2_ScN@C_80_ on Au(111) surface anchored to the Au(111) step edge (*V*_Bias_=2 V; *I*_Set_=200 pA); the arrow indicates the fullerenes closed packed direction coinciding with the Au‐[110] direction; the features of the reconstruction are altered at the interface. b) The field of view of the constant current topography image of the Dy_2_ScN@C_80_ monolayer on Au(111) (*V*_Bias_=1.5 V; *I*_Set_=600 pA) in which the electronic structure was investigated; the left upper inset shows the hcp arrangement of the single fullerenes and in the lower right inset the FFT of the image is presented. c) Four types of STS spectra measured for Dy_2_ScN@C_80_ monolayer on Au(111); asterisks mark features tentatively assigned to the fullerene LUMO. d) The average STS spectrum over the area shown in (b), revealing an effective gap of Δ_eff_=1.7±0.1 eV (gray curve) and the spectra averaged over two areas in (e) (red and blue curves). e) The dln(*I*)/dln(*V*)‐map measured at the HOMO level energy of *E*=−1.035 eV showing the spatial variations of the electronic structure attributed to the altered herringbone reconstruction at the interface to the fullerene monolayer. Average spectra of the regions in between the stripes (dark blue in (e)) and on the stripes (yellow/red in (e)) are presented in (d).

STM measurements revealed that at these evaporation conditions the surface of the substrate is covered with ca. 0.5 ML of fullerene molecules (Supporting Information, Figure S1). The endohedral fullerenes diffuse across the terraces of Au(111) and self‐assemble into closed monolayer islands of several 10×10 nm^2^ anchored to the edges of the substrate step (Figure 2 a). As already observed for other M_3_N@C_80_ fullerenes on Au(111),12a–12c Dy_2_ScN@C_80_ molecules are organized in a hexagonal close‐packed (hcp) structure with a lattice parameter of 1.15±0.05 nm (Figure 2 b). Fourier transformation of the topographic image gives Bragg peak positions proving a three‐fold symmetric periodic structure (Figure 2 b). On the bare part of the Au(111) substrate, the herringbone reconstruction typical for a clean Au(111) surface is observed (Figure 1 a). The reconstruction double stripes also appear on and across the monolayer islands (Figure 2 a,b), but the inter‐stripe distance and their course varies from that of unperturbed Au(111): The density of reconstruction stripes under the fullerene layer is reduced, whereas the Au(111) fcc‐like terminated areas in between the double stripes become wider in comparison to the pristine herringbone pattern. Thus, the formation of quasi‐epitaxial 4×4 superstructure of fullerenes on Au(111) fcc sites is enhanced and consequently the interface energy is reduced.12a, 12c, 15 The facts that the fullerenes show a sufficiently high mobility at RT to form monolayer islands and that the herringbone reconstruction is not fully lifted at the interface imply a comparably weak interaction of Dy_2_ScN@C_80_ molecules with the Au(111) surface.

The electronic structure of Dy_2_ScN@C_80_ on Au(111) was studied by scanning tunneling spectroscopy (STS). The spectra acquired in the field of view presented in Figure 2 b showed four somewhat different patterns (Figure 2 c) occurring with almost equal abundance. Presumably, they are caused by different tip position over fullerenes and by different adsorption geometries of the Dy_2_ScN@C_80_ molecules on the Au(111) surface.^16^ The noticeable difference between the splitting patterns demonstrates that the fullerene–fullerene and fullerene–substrate interactions are not negligible.^17^ Each spectrum type exhibits a well‐defined gap and distinct peaks corresponding to the occupied and unoccupied states of the fullerene molecule. DFT calculations show (see discussion below and in Supporting Information), that the density of fullerene states is rather high, and each peak in STS spectra corresponds to several fullerene orbitals. The HOMO‐derived state overlaps with other lower‐energy occupied states and form a peak at −(0.9–1) V. For the fullerene LUMO, DFT calculations predict a stand‐alone peak at the energy near +0.6 V above Fermi level, whereas other unoccupied orbitals are densely packed in the energy range above +1 V. Calculations also show that depending on the orientation of the endohedral cluster inside the fullerene versus the substrate, the orbital energies may vary within the range of 0.2–0.3 eV. Based on these predictions, we tentatively suggest that the LUMO‐derived states may corresponds to the features marked by asterisks in Figure 2 c. In the average spectrum of the whole studied area (Figure 2 d), these fine features are smeared out, leading to an effective energy gap of Δ_eff_=1.7±0.1 eV if the shoulder at +0.7 V is assigned to the LUMO or Δ_eff_=2.05±0.1 eV if only peak maxima are used.

The normalized differential conductance map (Figure 2 e) measured in the field of view shown in Figure 2 b illustrates that additional spatial variations of the electronic structure of the monolayer emerge from the formation of the Au(111) reconstruction at the interface with Dy_2_ScN@C_80_. At the HOMO energy of −1.035 eV below the Fermi level, regions of supposed Au(111) fcc‐like termination in between the topographically brighter double stripes in Figure 2 b are imaged in dark blue (Figure 2 e). They reveal a reduced local density of states in comparison to double stripe regions of the Au(111) hcp‐like and ridge‐like terminations, which correspond to the yellow–red region in Figure 2 e. This effect can be also seen as the difference of the HOMO‐derived peak heights in the STS spectra averaged over corresponding areas (Figure 2 d). Obviously, the adsorption sites of Dy_2_ScN@C_80_ molecules on Au(111) vary following the altered reconstruction from quasi‐epitaxial regions (in between the double stripes) to incommensurate double stripe areas, which affects the local electronic structure of the fullerene|metal interface.^18^ Note that the four types of STS spectra shown in Figure 2 c are found for both dark blue and bright red regions in Figure 2 e (Supporting Information, Figure S2).

The magnetic properties of Dy_2_ScN@C_80_ monolayers were studied by Dy‐*M*_4,5_ XMCD at the X‐Treme beam line at the Swiss Light Source, Paul Scherrer Institut.^19^ The magnetic field was kept parallel to the X‐ray beam in all measurements. The thin film of MgO (10–11 ML) on Ag(100) was grown by sublimation of Mg in O_2_ atmosphere (10^−6^ mbar) while keeping the substrate at 645 K. Dy_2_ScN@C_80_ evaporation conditions onto the Au(111) substrate were adopted from the ex situ studies described above. In situ characterization by STM confirmed formation of similar monolayer islands (Supporting Information, Figure S3). The same evaporation conditions were then used for the growth on Ag(100) and MgO|Ag(100), for which in situ STM characterization was not possible during the beamtime. The coverage of the Ag(100) and MgO|Ag(100) substrates by Dy_2_ScN@C_80_ was lower than for Au(111) (as estimated from XAS intensity), which ensures that all XMCD measurements were performed in the submonolayer regime. At the base temperature of the cryostat the temperature at the sample was near 2 K for the Au(111) crystal as estimated from separate measurements of Er(trensal)^20^ powder (Supporting Information, Figure S4). For Ag(100) and MgO|Ag(100) substrates the temperature may be slightly higher (up to ca 2.5 K).

**Figure 3:**
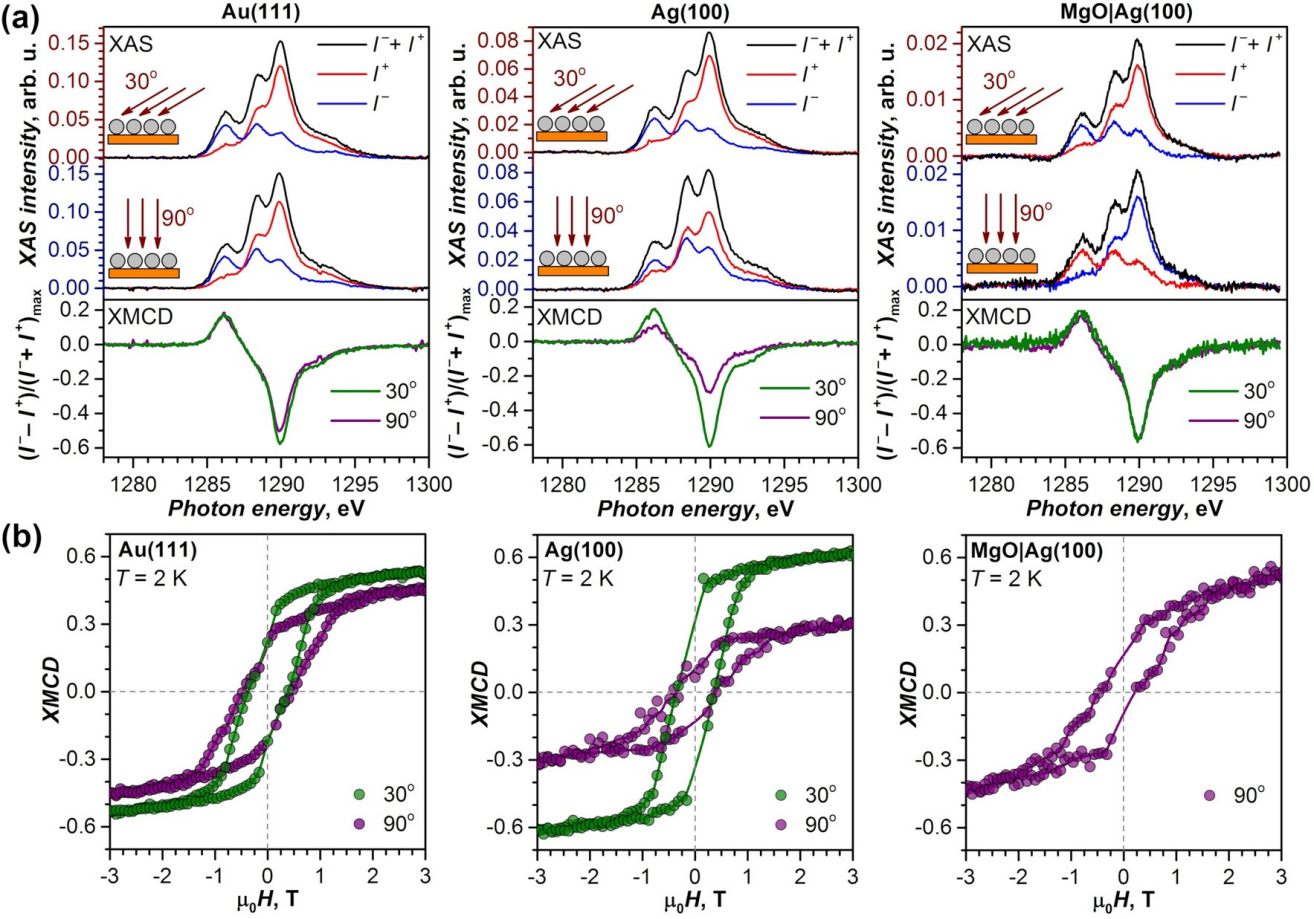
a) XAS and XMCD spectra of Dy_2_ScN@C_80_ sub‐monolayers on Au(111), Ag(100), and MgO|Ag(100) measured at 30° and 90° orientation of the X‐ray and magnetic field versus the surface; *T*≈2 K, *H*=6.5 T, only the Dy‐*M*_5_ edge is shown (see the Supporting Information for the whole Dy‐*M*_5,4_ range). X‐ray polarizations are denoted at *I* ^+^ and *I* ^−^, non‐polarized XAS is a sum of *I* ^+^ and *I* ^−^, and XMCD is their difference normalized to the XAS maximum. b) Magnetic hysteresis of Dy_2_ScN@C_80_ on Au(111), Ag(100), and MgO|Ag(100) measured by XMCD technique at *T*≈2 K, sweep rate 2 T min^−1^; dots are experimental values, and lines are added to guide the eye. For Au(111) and Ag(100), hysteresis measurements are shown for two angles of the X‐ray and magnetic field versus the surface.

**Figure 4:**
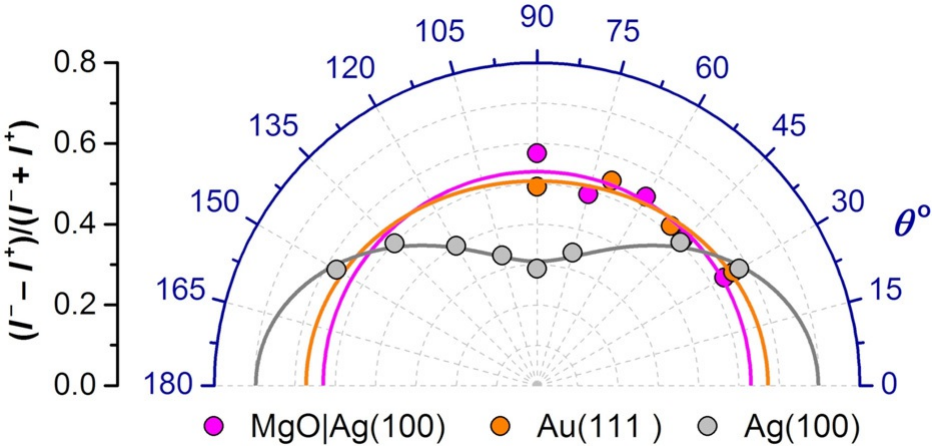
Angular dependence of XMCD asymmetry for the Dy_2_ScN@C_80_ submonolayer on Au(111), Ag(100), and MgO|Ag(100) measured at 1290 eV at *T*≈2 K. *θ* is the angle between X‐ray beam/magnetic field and the surface. Dots are experimental values, lines are fits with the function *C*_1_ cos^2^(*θ*)+*C*_2_.

Dy‐*M*_5_ XAS and XMCD spectra of Dy_2_ScN@C_80_ sub‐monolayers measured at *T*≈2 K in the magnetic field of 6.5 T are shown in Figure 3. The measurements at a different incidence of X‐rays and magnetic field show a noticeable difference between the substrates. On Au(111), the XMCD signal of Dy_2_ScN@C_80_ is slightly stronger in the grazing (30°) than in the normal (90°) incidence. On Ag(100), the difference between the two orientations is enhanced, with much stronger XMCD response at the grazing incidence. Finally, no difference can be seen between the spectra measured at 30° and 90° on the MgO|Ag(100) substrate. These results are further corroborated by the angular dependence of XMCD asymmetry (Figure 4), which is almost isotropic for Au(111) with a slight increase at smaller angles, isotropic for MgO|Ag(100) within the experimental uncertainty, but strongly anisotropic for the Ag(100) substrate.

As magnetic moments of Dy ions are strongly anisotropic and are locked to the direction of the Dy−N bonds (Figure 1 a), the angular dependence of XMCD essentially provides information on the orientation of the Dy_2_ScN clusters in the monolayers. The isotropic behavior of the XMCD signal on Au(111) and MgO|Ag(100) shows that the Dy_2_ScN cluster is randomly oriented in the Dy_2_ScN@C_80_ monolayers on these substrates. On the other hand, the anisotropy of the XMCD in the Dy_2_ScN@C_80_ monolayer on Ag(100) indicates that the endohedral cluster preferentially adopts a parallel orientation with respect to the surface. Parallel alignment of Dy_2_ScN was also observed for the Dy_2_ScN@C_80_ monolayer on Rh(111).7a

The magnetic moment of Dy^3+^ ions in Dy_2_ScN@C_80_ can be estimated using XMCD sum rules.^21^ For Dy_2_ScN@C_80_ on Au(111), the sum rule analysis gives the moment of 5.4±0.5 μ_B_ per Dy^3+^ ion at 90° and 6.1±0.5 μ_B_ at 30°. On Ag(100), the moment increases from 2.9±0.5 μ_B_ at 90° to 5.7±0.5 μ_B_ at 30°. These values are significantly smaller than 10 μ_B_, the ground‐state magnetic moment of Dy^3+^ ion in the axial ligand field. However, when magnetic moments are anisotropic, the measured moment corresponds to the projection of the magnetic field onto the easy axis. As a result, in powder samples of Dy_2_ScN@C_80_ with disordered Dy_2_ScN clusters, the apparent magnetic moment of Dy^3+^ ions is reduced to 5 μ_B_.^14^ The magnetic moments of Dy^3+^ ions in Dy_2_ScN@C_80_ on the Au(111) substrate are thus close to the value expected for the disordered cluster, which agrees with the lack of the angular dependence of the XMCD. For Dy_2_ScN@C_80_ on the Ag(100) substrates, the small magnetization perpendicular to the surface agrees well with the preferential in‐plane alignment of the endohedral cluster.

The XMCD signal at the Dy‐*M*_5_ edge was used to study magnetization curves of Dy_2_ScN@C_80_ monolayers. Magnetic hysteresis with a coercive field of ca 0.4 T is observed for Dy_2_ScN@C_80_ monolayers on all three substrates near 2 K (Figure 2 b). When temperature is increased to about 6 K, the hysteresis is not observed any more (Supporting Information, Figure S7). The asymmetric orientation of the Dy_2_ScN cluster is also reflected in magnetization curves measured at 30° and 90° on Au(111) and Ag(100) substrates (Figure 3 b). This width of the 2 K hysteresis is twice smaller than for the powder sample measured by SQUID magnetometry at a comparable sweep rate (Figure 1 b). A possible reason is the X‐ray‐induced demagnetization.^22^ Besides, changing polarity of the magnet during the field ramp takes 30 seconds, which also reduces the observed coercive field. Taking this into account, we conclude that there is no dramatic deterioration of the hysteretic behavior of Dy_2_ScN@C_80_ monolayers when compared to the bulk samples. Apparently, the fullerene cage provides sufficient protection for the endohedral magnetic cluster from the demagnetizing influence of metallic substrates. Earlier it was found that a thin layer of MgO dramatically increases the hysteresis of a TbPc_2_ monolayer5c, 23 and boosts the temperature of the magnetic bistability of Ho atoms up to 30 K.^24^ Recently, some of us showed that the improved SMM properties on MgO substrate are caused by its low phonon density of states, which leads to the dramatic reduction of the relaxation of magnetization via the Raman mechanism.^23^ Apparently, this is not the relaxation rate‐limiting factor for Dy_2_ScN@C_80_, and the use of the MgO layer does not lead to a noticeable improvement of the surface SMM behavior in comparison to metallic substrates. Thus, we conclude that the substrate plays an important role in the structural ordering of endohedral cluster, but has no strong influence on the SMM properties of adsorbed metallofullerenes. Therefore, the SMM performance of Dy_2_ScN@C_80_ monolayers is not limited by the interactions with the substrate.

**Figure 5:**
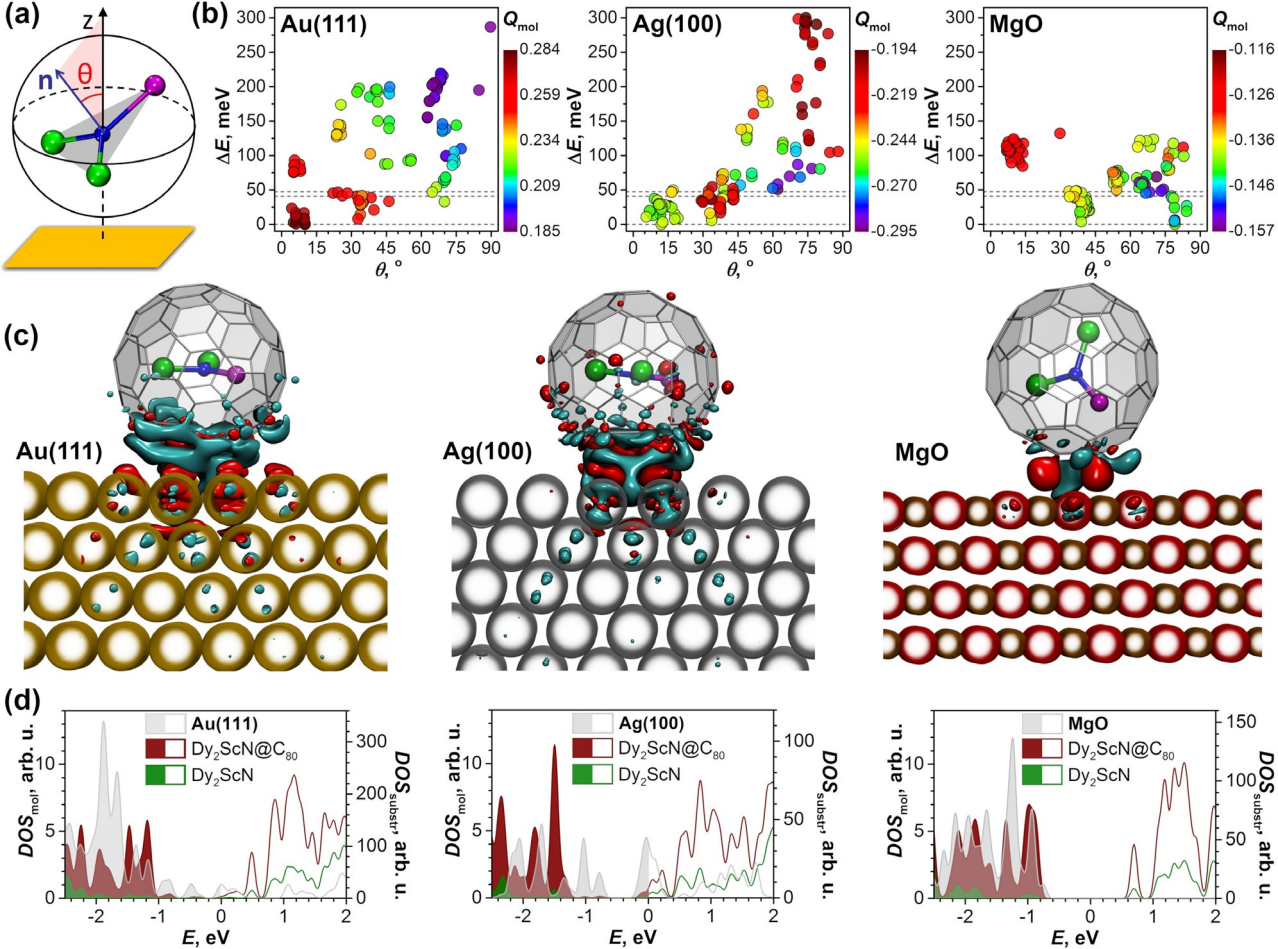
a) Definition of the cluster tilting angle *θ* as the angle between the axis *z* normal to the surface and the vector ***n*** radiating from the nitrogen atom perpendicular to the cluster plane; *θ*=0° and *θ*=90° correspond to the parallel and perpendicular alignment of the cluster versus the substrate, respectively. b) Relative energies of Dy_2_ScN@C_80_ conformers on Au(111), Ag(100), and MgO surfaces plotted versus *θ*; gray dashed lines mark the relative energies of the conformers of the free Dy_2_ScN@C_80_ molecule; color of the dots codes the net charge of Dy_2_ScN@C_80_ molecules (*Q*_mol_) on a surface. c) Isosurfaces of the difference electron density for Dy_2_ScN@C_80_ molecule on different substrates (red color marks regions with the increased electron density, whereas cyan corresponds to the depletion of the density; all three systems are plotted at the same isovalue). d) DFT‐computed density of states (DOS) near the Fermi level projected onto Dy_2_ScN@C_80_ molecule and Dy_2_ScN cluster states (dark red and green, respectively; the axis is denoted as DOS_mol_) and the substrate‐projected DOS (DOS_substr_, semi‐transparent gray). Note that the DOS_mol_ scale is the same, whereas the DOS_substr_ scale varies in each part of the figure.

For a deeper insight into the fullerene‐substrate interactions, DFT calculations of the Dy_2_ScN@C_80_ molecule placed on Au(111), Ag(100), and MgO surfaces were performed at the PBE‐D level with PAW 4f‐in‐core potentials using the VASP 5.0 code.^25^ As the Dy_2_ScN cluster may adopt different orientations inside the carbon cage, it is important to have a comprehensive sampling of possible structural configurations. Using recently proposed Fibonacci sphere sampling,^26^ 120 initial configurations with different orientations of the Dy_2_ScN cluster were generated for a fullerene molecule on each substrate, and their structures were optimized. For an isolated Dy_2_ScN@C_80_ molecule this approach gave only three unique conformers with the relative energies of 0, 41, and 47 meV. But a substantially different situation is found for Dy_2_ScN@C_80_ molecule on a substrate (Figure 5 a,b). Calculations did not reveal any particularly stable conformation but rather predicted a multitude of conformers, which are likely to coexist under experimental conditions within a certain energy cut‐off. The energies of the on‐surface optimized conformers are spread in the range of 300 meV for Au(111) and Ag(100) substrates and 170 meV for the MgO substrate. It should be emphasized that these conformer distributions are predicted for a single Dy_2_ScN@C_80_ molecule in the absence of other fullerene neighbors. Due to the incommensurate lattice parameters of the substrates and the fullerene layer, calculations of the Dy_2_ScN@C_80_ monolayer are not feasible at this time. It can be anticipated that the intermolecular interactions should also affect the energetics of the cluster orientations and electronic properties,^26^ but the influence of the metallic substrate is expected to be considerably stronger.

For Dy_2_ScN@C_80_ on Au(111) and Ag(100), the lowest‐energy conformers are grouped at *θ*=5–30°, which corresponds to nearly parallel orientations of the cluster to the substrate. These results agree well with the observation of the in‐plane ordering of the Dy_2_ScN cluster on the Ag(100) surface, but do not fully capture the experimental finding of the weaker ordering effect on the Au(111) substrate. Presumably, the Au(111) surface reconstructions may decrease the ordering of the cluster in adsorbed fullerenes, but computational description of such effects is not feasible at this moment. For the MgO substrate, calculations show the grouping of the most stable conformers near the angles of 35–40° and 75–85°, but the overall energy spread of the conformers is smaller than on metals. Although XMCD measurements are performed 2 K, it is reasonable to expect that the distribution of the conformers in the experimental samples should be different from the equilibrium for 2 K. When the sample is cooled down from room temperature, at certain point above the base temperature the angular distribution of the conformers will be frozen. Effectively, this situation can be modelled by considering conformers within a certain energy cut‐off. We simulated XAS and XMCD spectra of different conformers with MULTIX code^27^ (Supporting Information, Figure S8) and found that when an arbitrary energy cut‐off of 50 meV is used, the simulations reproduce the experimentally found lack of the ordering on MgO|Ag(100) and preferential in‐plane alignment on Ag(100).

The PBE‐D binding energy of the fullerene molecule to the surface is 2.91 eV for Au(111), 2.53 eV for Ag(100), and 1.43 eV for MgO (Table 1). The main contributions are from the dispersion interactions (*E*_disp_).25e Deformation energy *E*_def_ necessary to distort the structures of the isolated Dy_2_ScN@C_80_ molecule and the substrate to those they adopt in the interacting system, is found to be 366 meV for Au(111), 157 meV for Ag(100), and 39 meV for MgO, to which fullerene contributions are 132, 46, and 12 meV, respectively. The remaining terms of 0.64 eV for Au(111), 0.47 eV for Ag(100), and −0.13 eV for MgO are due to the electronic contribution *E*_Coul/cov_, which includes both Coulomb and covalent terms. Though 4–5 times smaller than dispersion, these interactions are responsible for the changes in the electronic structure at the interface and hence are considered further in more details. Note also that the variation of the relative energy for different cluster orientations (Figure 5 b) is mainly caused by the changes in *E*_Coul/cov_, whereas *E*_disp_ remains almost identical for the whole conformer set, and variations in *E*_def_ are considerably smaller (Supporting Information, Table S1).

**Table 1:** Contributions to the fullerene‐substrate binding energy [eV] and molecular and cluster charges for Dy_2_ScN@C_80_ adsorbed on different surfaces.

*E* ^[a]^	Au(111)	Ag(100)	MgO
*E*_tot_	2.909	2.534	1.433
*E*_disp_	2.634	2.223	1.603
*E*_def_	−0.366	−0.157	−0.039
*E*_Coul/cov_	0.641	0.468	−0.131
			
*Q*_mol_	+0.28	−0.25	−0.14
*Q*_cluster_	+3.85	+3.84	+3.85

[a] Total fullerene‐substrate interaction energy *E*_tot_ is the energy difference between the fullerene adsorbed on the substrate and separated fullerene molecule and the substrate in their optimized structures; positive sign indicates stabilizing interaction. *E*_tot_ is partitioned into dispersion, deformation, and Coulomb/covalent contributions: *E*_tot_=*E*_disp_+*E*_def_+*E*_Coul/cov_. *E*_tot_, *E*_disp_ and *E*_def_ can be computed independently, which allows estimation of *E*_Coul/cov_.

Interaction of a fullerene molecule with the substrate leads to a change in the electronic distribution of both. The net effect of this redistribution can be evaluated via the charge of the fullerene molecule (*Q*_mol_) accumulated on the surface. Calculations of atomic charges with the Bader code^28^ showed that Dy_2_ScN@C_80_ transfers 0.2–0.3 *e* to the Au(111) substrate, but acquires a negative charge of −(0.2–0.3) *e* on Ag(100) and −(0.09–0.14) *e* on MgO. Importantly, despite the considerable variation of the net fullerene charge in dependence on the substrate and the cluster orientation (Figure 5 b), the charge of the Dy_2_ScN cluster in adsorbed molecules is virtually the same as in the isolated Dy_2_ScN@C_80_ molecule and varies by less than ±0.05 *e* (compare to the 0.57 *e* variations of *Q*_mol_). The role of the Faraday cage that the fullerene plays for the endohedral cluster7c, 29 may be the reason why SMM properties of Dy_2_ScN@C_80_ are weakly affected by the substrate.

A closer look into the interfacial charge transfer is provided by the difference electron density Δ*ρ* obtained by subtraction of the electron density of separately computed Dy_2_ScN@C_80_ molecule and a substrate from the electron density of the whole system. Visualization of Δ*ρ* in Figure 5 c shows that the charge redistribution is restricted to the interfacial region where the fullerene molecule contacts the substrate. The largest part of the carbon cage as well as the endohedral cluster are only weakly affected. When Dy_2_ScN@C_80_ is adsorbed onto the Au(111) surface, density depletion and accumulation regions are formed near the fullerene and near/at the upper gold metal atoms, respectively. For the fullerene on the Ag(100) surface, the regions of the density accumulation and depletion are intertwined in a complex manner. The spatial extension of the density affects at least two layers of Au and Ag metal atoms and small changes can be seen down to the fourth layer. For the fullerene on the MgO, the difference density has the least extended profile, and changes in the electron density are visible only in the upper layer of the substrate.

The spatial extension of Δ*ρ* correlates with the angular dependence of the relative energy of the Dy_2_ScN@C_80_ conformers. Apparently, the Dy_2_ScN cluster tends to avoid the parts of the fullerene π‐system interacting with the metallic surface. This can be best achieved in the parallel configuration of the Dy_2_ScN cluster, in which all three metal atoms do not interact with the surface‐perturbed parts of the fullerene cage. For the MgO substrate, the situation is different because the ionic substrate has strongly inhomogeneous charge distribution, and here the electrostatic interactions between the substrate and the carbon cage play an important role. As the fragments of the fullerene cage coordinated by the endohedral metals have pronounced variation of the electrostatic potential distribution,^26^ the orientation of the fullerene molecule towards the substrate by such fragments may become energetically favorable.

Interactions with the substrate may also affect the electronic structure of the adsorbed fullerene. Comparison to the density of states (DOS) of the isolated Dy_2_ScN@C_80_ molecule (Supporting Information, Figure S12) shows that interaction with the MgO has only a minor effect on the fullerene‐projected DOS. The highest‐occupied states are dominated by the carbon cage, whereas the lowest‐energy unoccupied states have noticeable contributions from the endohedral cluster. The LUMO of the fullerene has a distinct peak in the DOS at 0.85 eV above the Fermi level. The DFT‐predicted gaps for the isolated Dy_2_ScN@C_80_ molecule and the molecule on the MgO are 1.65 and 1.63 eV, respectively. The interaction with the Au(111) surface results in more pronounced changes of the fullerene DOS (Figure 5 d). The HOMO‐ and LUMO‐derived peaks can be seen in the DOS at −1.09 eV and 0.59 eV, respectively. The gap between the LUMO peak and higher‐energy unoccupied states is reduced substantially. Furthermore, new states appear in the gap between the HOMO and LUMO. They originate from the hybridization with the metal bands and can be identified as surface states. Thus, the weak features inside the gap in the experimental STS spectra of Dy_2_ScN@C_80_ on Au(111) (Figure 2 c) may have its origin here. Finally, the most pronounced changes of the DOS are found for the fullerene on Ag(100). Here the LUMO‐derived feature is not a single peak anymore but is split into several components at the Fermi energy. Thus, the LUMO of Dy_2_ScN@C_80_ is contributing strongly to the surface states and becomes partially occupied.

## Conclusion

Realization of magnetic bistability in monolayers of SMMs in contact with an electrode is a necessary step towards making use of the single‐molecule nature of their magnetism in spintronic devices. In this work we showed that Dy_2_ScN@C_80_ fulfills this criterion. XMCD studies of the Dy_2_ScN@C_80_ submonolayers on Au(111), Ag(100), and MgO|Ag(100) revealed a distinctive influence of the substrates on the ordering of the endohedral cluster. On Ag(100), the Dy_2_ScN units are preferentially aligned parallel to the surface, on Au(111) there is only a slight preference of the parallel alignment, whereas on MgO|Ag(100) no ordering is found at all. However, despite the strong influence of the surface on the structural ordering, the magnetic behavior of Dy_2_ScN@C_80_ molecules does not show a noticeable dependence on the substrate. Magnetic hysteresis with the coercivity of ca 0.4 T is found near 2 K in submonolayers of Dy_2_ScN@C_80_ on all three substrates. DFT calculations showed that the charge redistribution at the metal–fullerene interface is confined within the contact region. The electron transfer affects only the carbon cage, whereas the charge state of the endohedral cluster remains intact. Thus, the fullerene acts as a Faraday cage protecting the electronic and magnetic properties of the endohedral species on conducting substrates.

## Conflict of interest

The authors declare no conflict of interest.

## Supporting information

As a service to our authors and readers, this journal provides supporting information supplied by the authors. Such materials are peer reviewed and may be re‐organized for online delivery, but are not copy‐edited or typeset. Technical support issues arising from supporting information (other than missing files) should be addressed to the authors.

Supplementary
