# Supplementary material for: Substrate‐Independent Magnetic Bistability in Monolayers of the Single‐Molecule Magnet Dy2ScN@C80 on Metals and Insulators
Source: Angew Chem Int Ed Engl. 2020 Jan 24;59(14):5756–64. doi: 10.1002/anie.201913955 (PMC7155138; doi:10.1002/anie.201913955)
Supplement: Supplementary file 1 — Supplementary [file ANIE-59-5756-s001.pdf]

Angewandte Chemie
Eine Zeitschrift der Gesellschaft Deutscher Chemiker

Supporting Information

Substrate-Independent Magnetic Bistability in Monolayers of the Single-Molecule Magnet $Dy_2ScN@C_{80}$ on Metals and Insulators

Denis S. Krylov[+], Sebastian Schimmel[+], Vasilii Dubrovin[+], Fupin Liu, T. T. Nhung Nguyen, Lukas Spree, Chia-Hsiang Chen, Georgios Velkos, Claudiu Bulbucan, Rasmus Westerström, Michał Studniarek, Jan Dreiser, Christian Hess, Bernd Büchner, Stanislav M. Avdoshenko,* and Alexey A. Popov**

anie_201913955_sm_miscellaneous_information.pdf

Table of Contents

Experimental details	2
Additional STM data	3
Estimation of the sample temperature from Er-trensal measurements	5
XAS and XMCD spectra	6
Additional hysteresis curves	7
Simulations of XAS and XMCD spectra	8
Additional computational results	9
Author contributions	15
References	15

Experimental details

Fullerene preparation: $Dy_2ScN@C_{80}$ was synthesized by arc-discharge synthesis and purified by high-performance liquid chromatography (HPLC) as described in Ref. [1]. After HPLC separation, $Dy_2ScN@C_{80}$ was washed with acetone and hexane and then transferred to the crucible for evaporation by drop-casting from toluene.

$Dy_2ScN@C_{80}$ film growth on Au(111) and STM measurements: Before the fullerene deposition, the single-crystalline gold substrate was prepared by cycles of Ar-ion sputtering ($E_{Ar^+} = 1$ keV, t = 20 min) and subsequent annealing (t = 120 min; $T \approx 820$ K) to ensure its cleanliness and formation of extended terraces bounded by step-edges of monoatomic height. $Dy_2ScN@C_{80}$ was then deposited onto the Au(111) surface using a home build evaporator with the distance between the crucible and a substrate reduced to 5 cm. Typical evaporation conditions are then 20 minutes at 450–460 ºC, but a longer time may be required when the amount of fullerenes in the crucible is low. The substrate was kept at room temperature during deposition.

After the evaporation, the sample was in situ studied by room temperature STM. Topography of the monolayers was studied by constant current imaging mode. In order to further investigate the electronic properties of the monolayer configuration of $Dy_2ScN@C_{80}$ on Au(111), after obtaining the topography image of Fig. 2b a full spectroscopic map, by means of I(V)-spectra taken in a grid of 128x128 pixels, was acquired in the same field of view. Each measured spectrum contains data points of 256 equidistant steps over a voltage range of +/- 2 V. The access to the differential conductance, which is supposed to be proportional to the local density of states was in a first step attained by numerical differentiation. Due to the setpoint (V_{Bias} = 2 V, I = 600 pA), towards low bias voltages the signal is reduced and because of the comparatively high bias voltages the voltage dependence of the tunneling matrix element cannot be neglected. Therefore a second mathematical step that is commonly used in the field of STS on molecules was conducted. In order to reduce the influence of the voltage dependent tunneling matrix element and thus relatively enhance the dI/dV-signal towards zero bias it was normalized via dividing by the corresponding I/V-ration and hence the expression dlnI/dlnV or the normalized dI/dV was obtained. Great care was taken to avoid dividing by zero. Due to edge effect of the numerical treatments the presented spectra were reduced to the reliable unaffected +/- 1.7 V range of interest. The averaged I(V)-spectra of the four different types and the associated normalized dI/dV-spectra are shown in Fig. S3.

XMCD measurements and sample preparation: The surfaces of Au(111) and Ag(100) single crystal substrates were prepared by repeated Ar^+-sputtering and annealing cycles. The thin film of MgO on Ag(100) was grown by sublimation of Mg in O_2 atmosphere (10^{-6} mbar) while keeping the substrate at 645 K, the film thickness determined by XAS at the Mg-K edge was in the range of 10–11 monolayers.

$Dy_2ScN@C_{80}$ evaporation conditions onto the Au(111) substrate were adopted from the *ex situ* studies. The same evaporation conditions were then used for the growth on Ag(100) and MgO|Ag(100), for which *in situ* STM characterization was not possible during the beamtime. Due to the gradually reducing amount of fullerenes in the crucible, the coverage of the Ag(100) and MgO|Ag(100) substrates by $Dy_2ScN@C_{80}$ was lower than for Au(111) (as estimated from XAS intensity), which ensures that all XMCD measurements were performed in the submonolayer regime.

For $Dy_2ScN@C_{80}$ on Au(111) and Ag(100) substrates, Dy-$M_{4,5}$ XAS spectra were measured in the 1265-1350 eV energy range (Fig. S5). For the MgO|Ag(100) substrate, the measurement of XAS at the Dy-M_4 edge was not possible due to the strong Mg-K XAS signal of the MgO substrate in the same energy range.

XAS spectra measured in two polarizations were first normalized to the pre-edge absorption. After this normalization, the background was subtracted giving the XAS intensity scale used in the manuscript (Figure 3 and S6). To ensure correct comparison of XMCD intensities for the measurements at difference incidence angles, the XMCD values were divided by the maximal XAS intensity at each angle.

DFT calculations: DFT calculations of the $Dy_2ScN@C_{80}$ molecule placed on Au(111), Ag(100), and MgO surfaces were performed at the PBE-D level with PAW potentials using the VASP 5.0 code.[2] The 4f-in-core potentials allow DFT calculation of Dy-containing molecules without explicit treating of 4f electrons at the DFT level. Substrates for Au(111) and Ag(100) were modeled by six atomic layers with the unit cell of 14.425 × 14.425 × 40.0 Å (90°, 90°, 60°) for Au(111), and 14.46 × 14.46 × 40 Å (90°, 90°, 90°) for Ag(100). MgO(100) was modeled by 4 atomic layers with the unit cell of 12.636 × 12.636 × 30.0 Å (90°, 90°, 90°). The distance of 40 Å (Au, Ag) and 30 Å (MgO) between the replicas in z-direction ensures the absence of interactions between them. In the course of a structure optimization, both the fullerene and the substrate atomic positions were allowed to refine. The numerical settings used in these calculations ensured the uncertainty of the relative energies of less than 1–2 meV. Due to the large size of the unit cell, computations were performed only in the Γ-point. Density of states plots were obtained from the energy spectrum in the Γ-point convoluted with the Gaussian function of 0.05 eV.

XAS simulations: Theoretical XAS and XMCD spectra for the Dy-$M_{4,5}$ edge were simulated based on the point charge model as implemented in MULTIX code.[3] In these calculations, the Dy atoms assumed to be Dy(III) ions surrounded by point charges. These charges were derived from DFT-computed electronic densities using Bader code[4] for each of 120 optimized conformers of $Dy_2ScN@C_{80}$ molecule on three considered substrates (Ag, Au and MgO). A set of semiempirical parameters was used to match experimental M_4-M_5 splitting (spin-orbital couplings of 0.95 for core and of 0.85 for valence states were taken as recommended values) and the energy spread of the M_5 edge (on-site) was affected by Coulomb shift of 0.75, see ref. [3] for full details. To account for experimental broadening, the theoretical spectra were convoluted with the Gaussian function (σ = 0.5 eV). The magnetic field strength and modeling temperature were set to the experimental values of 6.5 T and 2.0 K, respectively. In all simulations, the X-ray beam is collinear to the applied magnetic field and was considered in two relevant directions, one perpendicular to the surfaces and one with the tilting angle of 30º. The spectra were computed for each conformer separately and then averaged over the set of conformers within the energy cut-off of 50 meV or over the whole set of conformers.

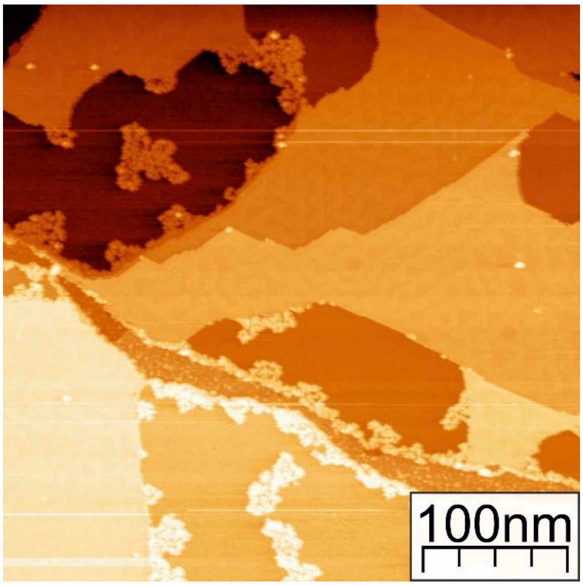

Figure S1. Constant current topography image of $Dy_2ScN@C_{80}$ on Au(111) (V_{Bias} = 2 V; I_{Set} = 500 pA, room temperature). The fullerene-monolayer-islands cover the substrate surface to ca. 50%

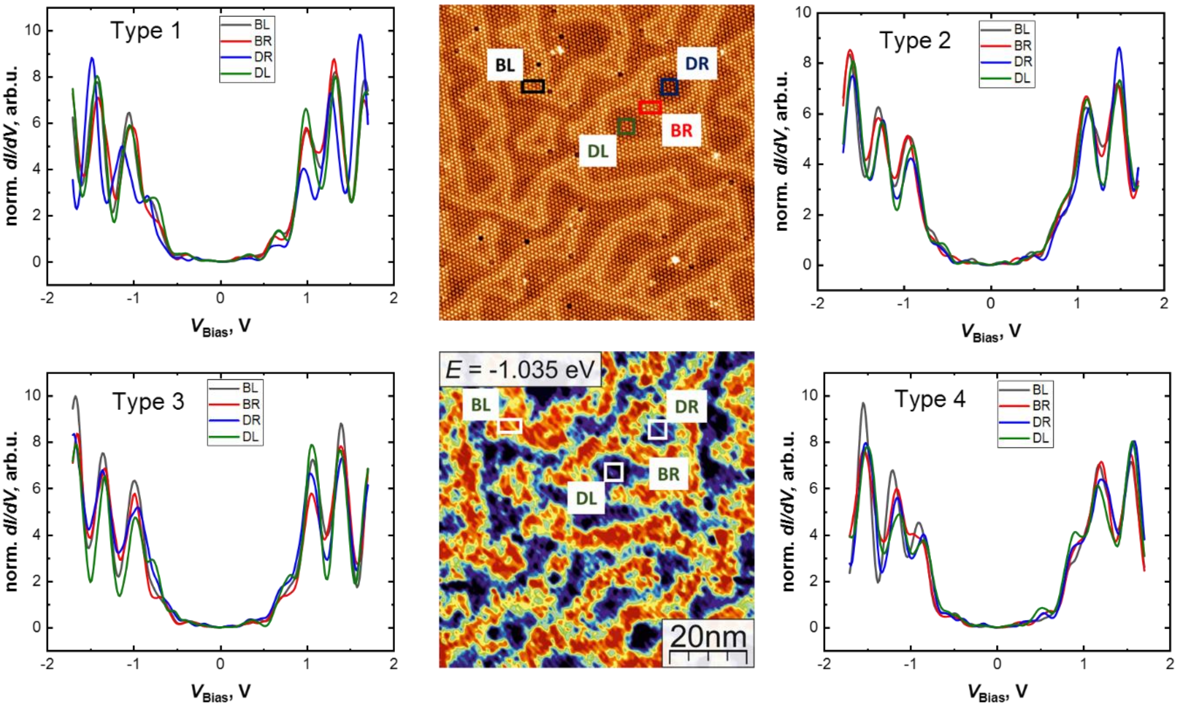

Figure S2. *Middle:* constant current topography image of the $Dy_2N@C_{80}$ monolayer on Au(111) and the dln(I)/dln(V)-map measured at the HOMO-level energy of $E = -1.035$ eV in the same field of view. Colored (or white) rectangles show two dark and two bright areas (marked as BL, DL, BR, DR), for which STS spectra are compared. *Left and right*: four types of STS spectra occurring in each highlighted area (compare to Fig. 2c; color code of the curves corresponds to the color of rectangles in the upper middle topography image).

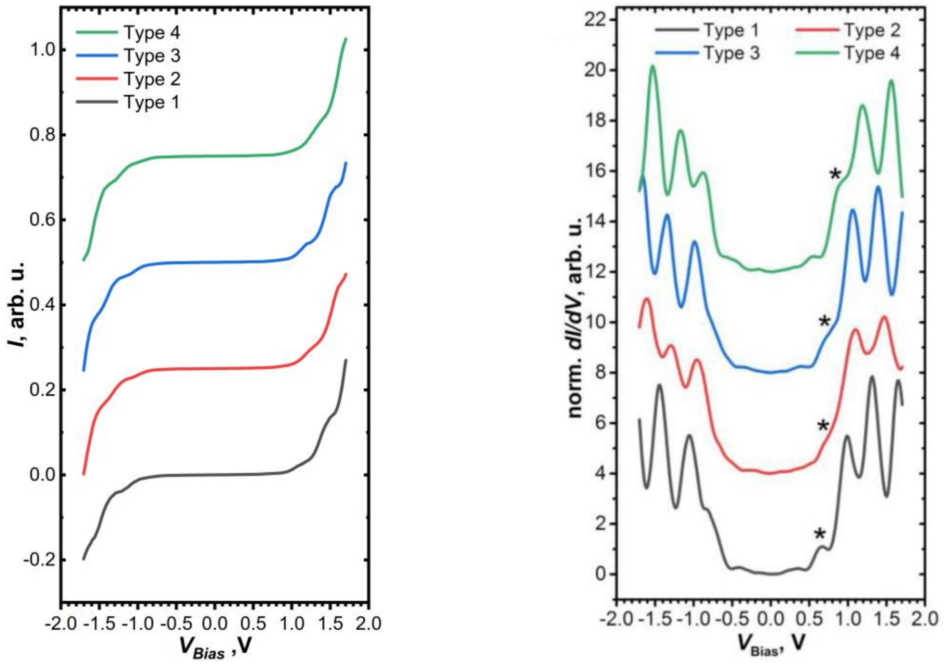

Figure S3. Comparison the *I(V)*-spectra (*left*) and the corresponding norm. *dI/dV*-spectra (*right*).

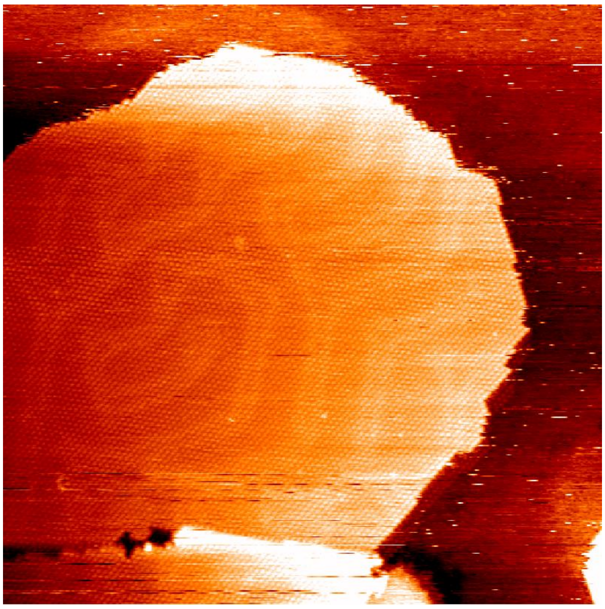

Figure S4. Constant current topography image of just evaporated $Dy_2ScN@C_{80}$ island on Au(111) measured *in situ* before XAS studies.

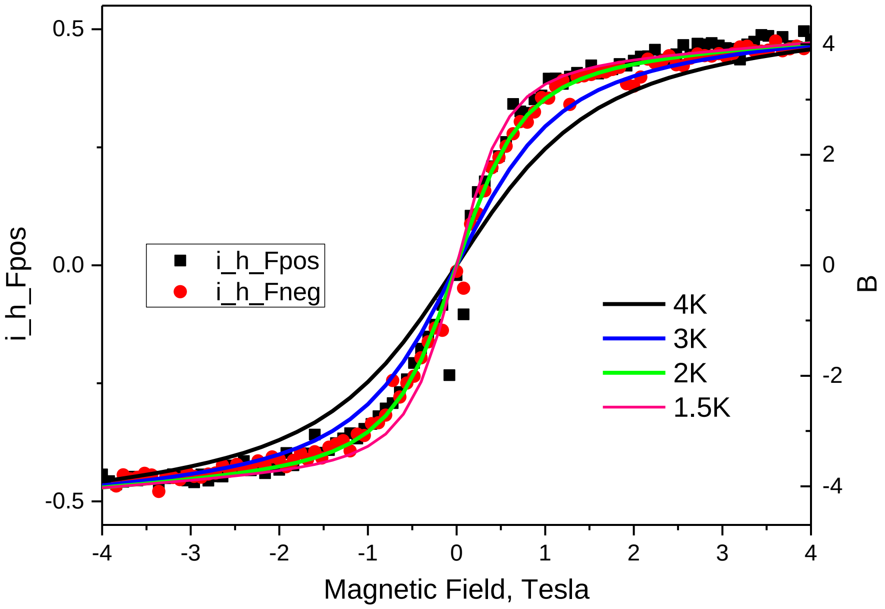

Figure S5. Magnetization curves of Er-trensal powder on Au(111) crystal at the base temperature compared to the simulated curves for different temperatures.

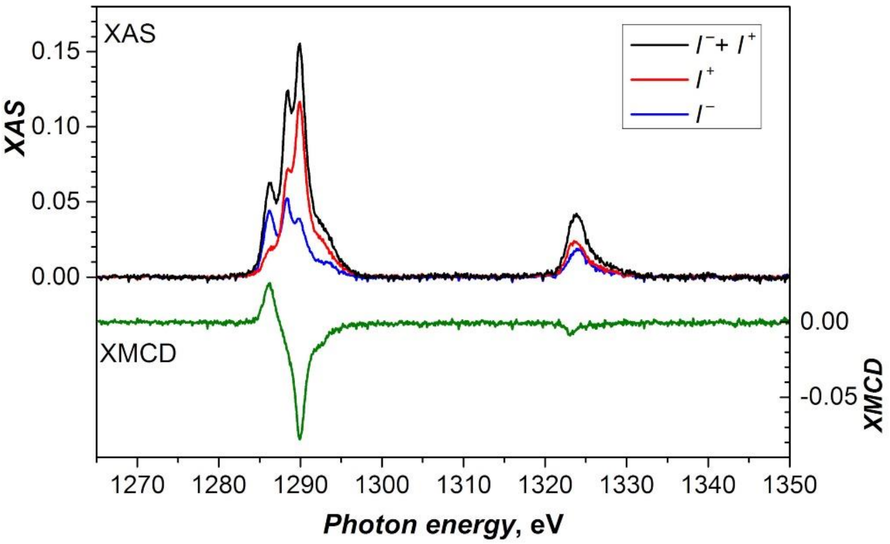

Figure S6a. XAS (top) and XMCD (bottom) spectra of $Dy_2ScN@C_{80}$ adsorbed on Au(111) at the Dy-$M_{4,5}$ edge, normal incidence.

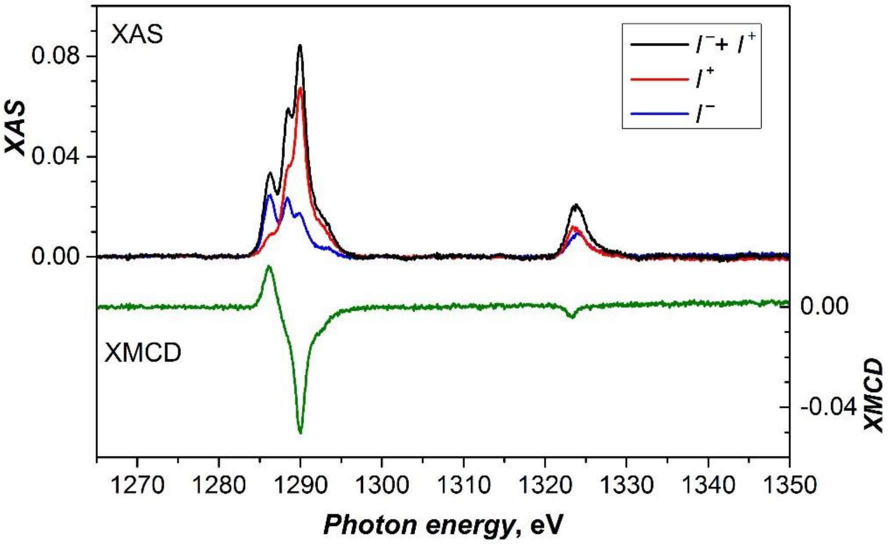

Figure S6b. XAS (top) and XMCD (bottom) spectra of $Dy_2ScN@C_{80}$ adsorbed on Ag(100) at the Dy-$M_{4,5}$ edge, grazing incidence.

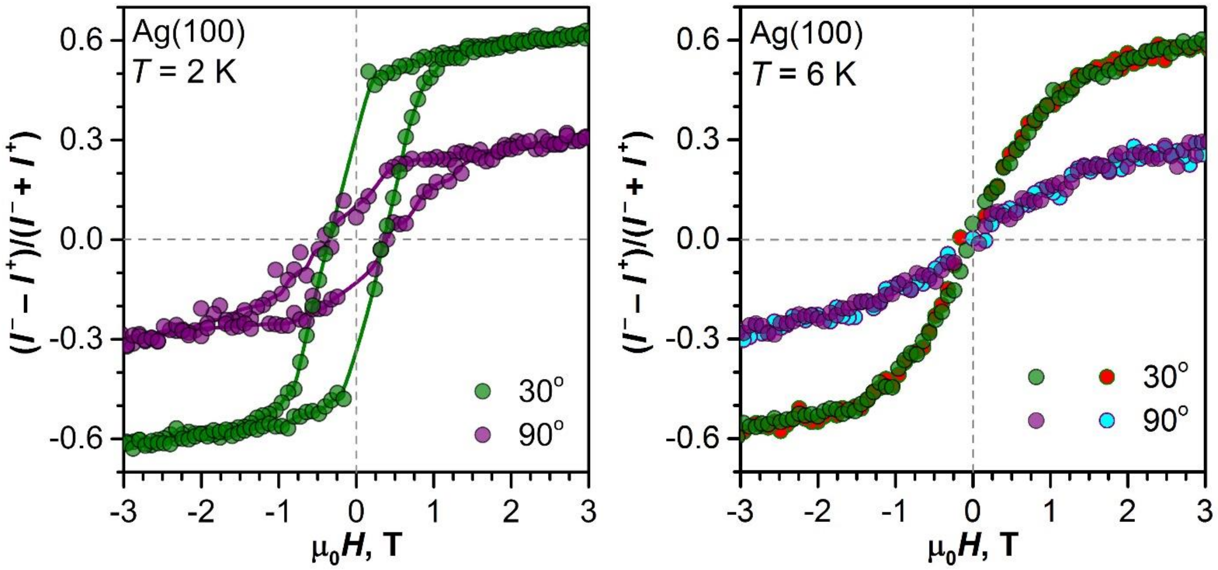

Figure S7. Magnetization curves of $Dy_2ScN@C_{80}$ on Ag(100) measured by XMCD at ca 2 K (left) and ca 6 K (right) in two orientations of the field and X-ray beam versus the surface. For 6 K measurements, field sweeps in different directions are plotted in different color. Magnetic hysteresis at 6 K is closed. Stabilization of the temperature between 2 K and 6 K was not possible with the type of cryostat used in the measurements. Note that the real temperature on the sample may be slightly different (ca 0.5 K higher).

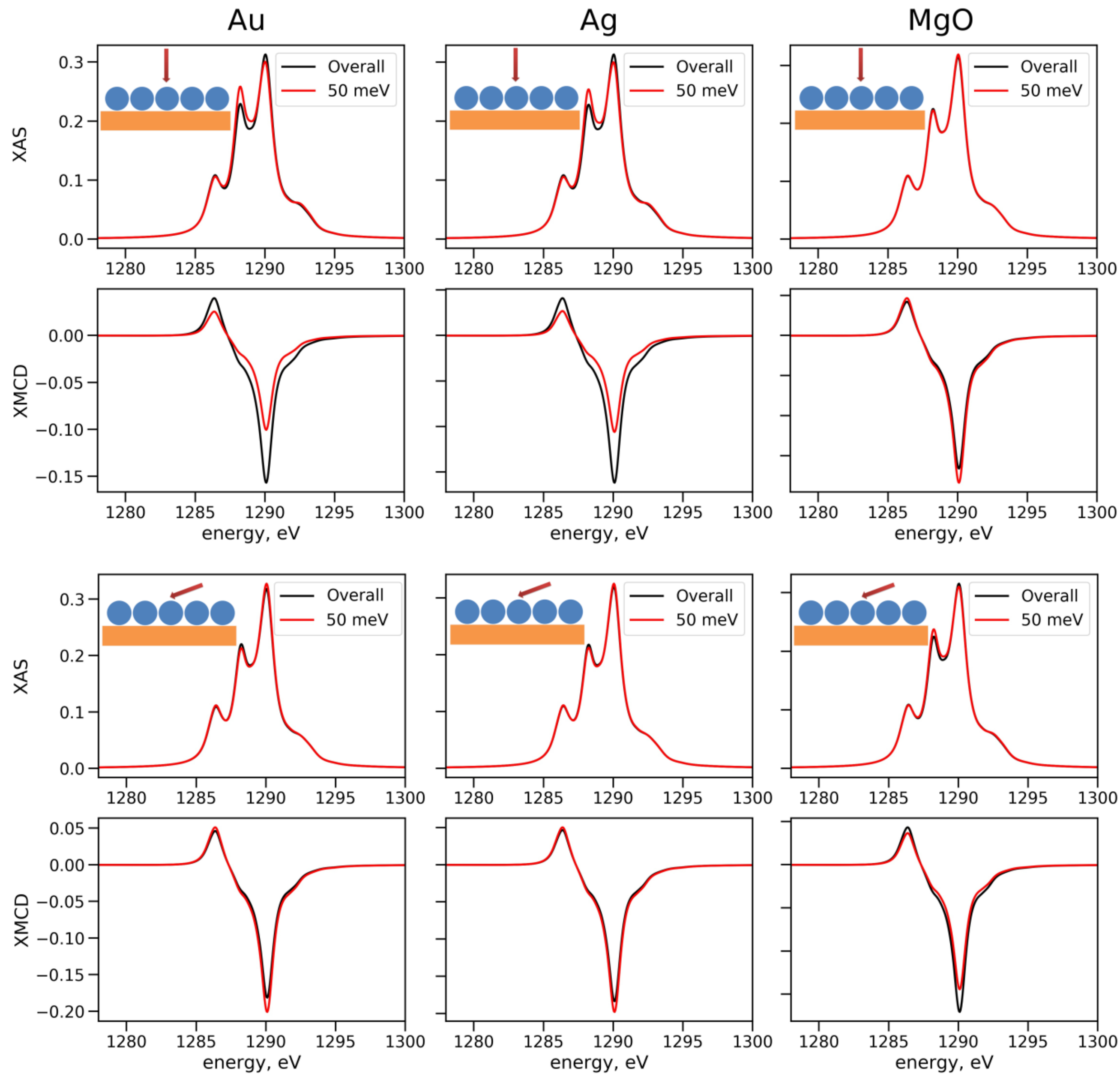

Figure S8. Averaged theoretical XAS and XMCD spectra for the whole set of 120 DFT-optimized conformations of $Dy_2ScN@C_{80}$ molecule on Au, Ag, and MgO substrates compared to the averaged spectra for a reduced set of conformes within the relative energy window of 50 meV. Two orientations of the beam versus the substrate are 90º and 30º. Interestingly, the spectra at normal incidence appear to be much more dependent on the conformer distribution than the spectra taken near grazing incidence.

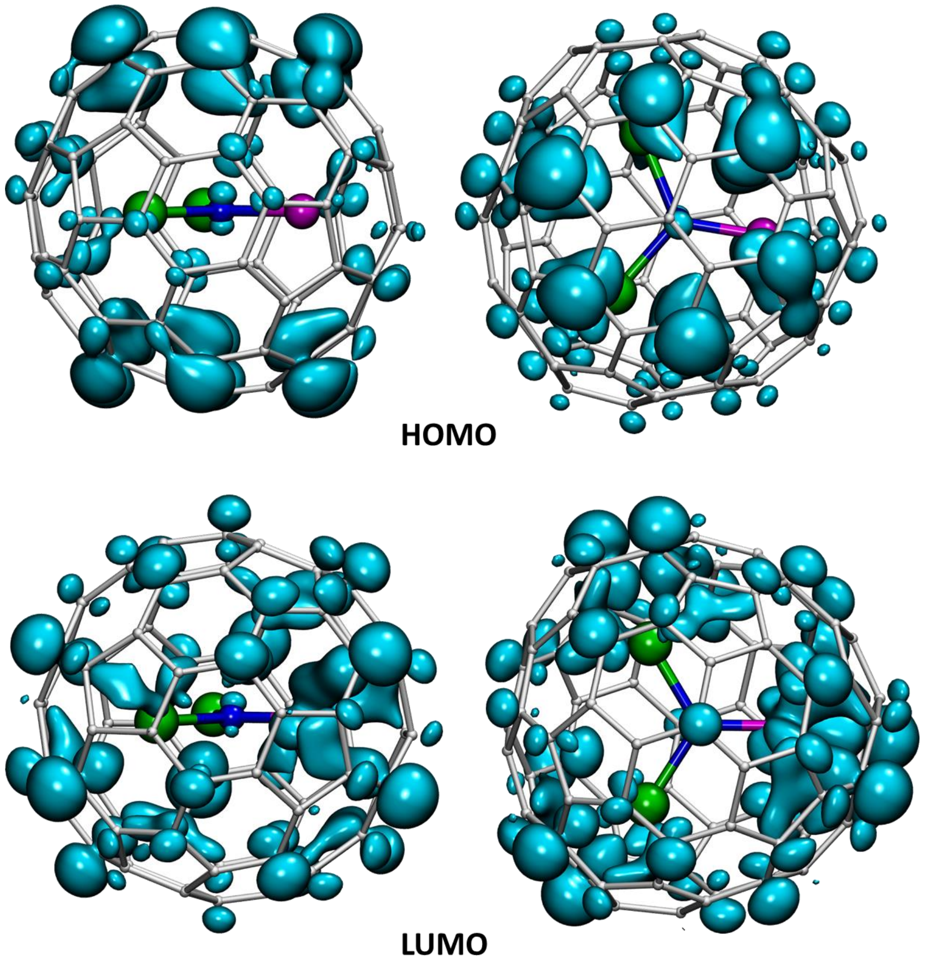

Figure S9. Orbital density isosurfaces for the HOMO and LUMO of the isolated $Dy_2ScN@C_{80}$ molecule.

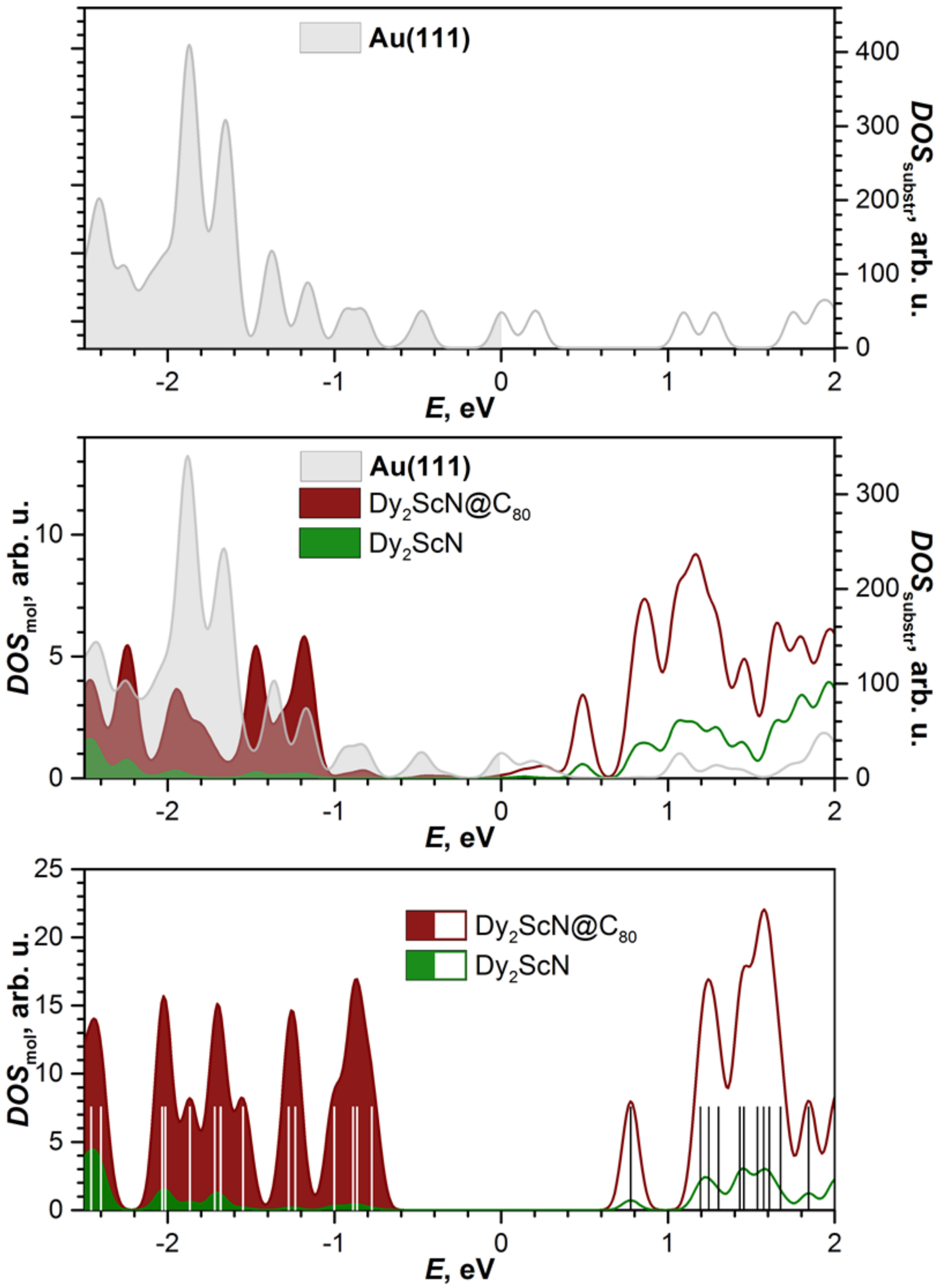

Figure S10. DFT-computed DOS of the bare Au(111) substrate (top), isolated $Dy_2ScN@C_{80}$ molecule (bottom) and $Dy_2ScN@C_{80}$ molecule on Au(111) (middle). Vertical bars show orbital energies of the isolated molecule. Fermi level of the isolated molecules is set to $(E_{HOMO}+E_{LUMO})/2$. Note that even the isolated fullerene molecule has rather high density of states. For the HOMO energy range, several fullerene states overlap thus forming a broad and intense feature. The LUMO is a stand-alone feature, which is preserved after adsorption. It is possible that the LUMO-derived band in STS spectra corresponds to the low-intensity peak or shoulder near 0.6 eV, whereas the peak near 1 eV corresponds to several fullerene states of higher energy.

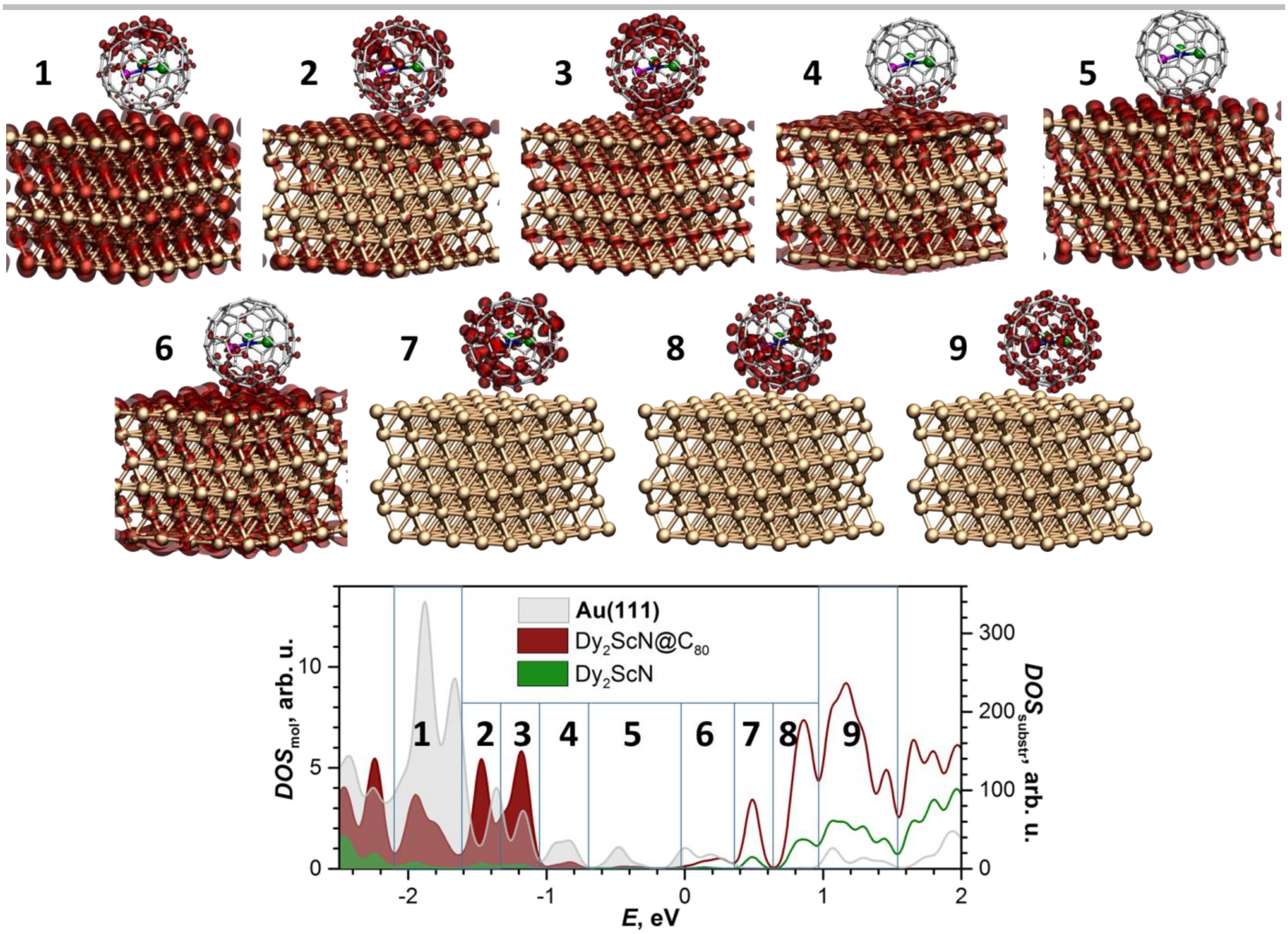

Figure S11. DFT-computed electron density isosurfaces of $Dy_2ScN@C_{80}$ on Au(111) (the lowest-energy conformer) corresponding to the selected energy ranges numbered in the DOS plot at the bottom of the figure. Note that the Au(111) states at low energies above and below Fermi level are mainly surface states of the substrate.

Table S1. Contributions to the fullerene-substrate binding energy for $Dy_2ScN@C_{80}$ adsorbed on different surfaces in the lowest energy (“min”) and the highest energy (”max”) conformers, all values in eV.

	Au(111) min	Au(111) max	Ag(100) min	Ag(100) max	MgO min	MgO max
E_{tot}	2.909	2.610	2.534	2.150	1.421	1.268
E_{disp}	2.634	2.637	2.223	2.151	1.583	1.469
E_{def}	0.366	0.318	0.157	0.114	0.026	0.023
$E_{Coul/cov}$	0.641	0.291	0.468	0.113	−0.136	−0.178

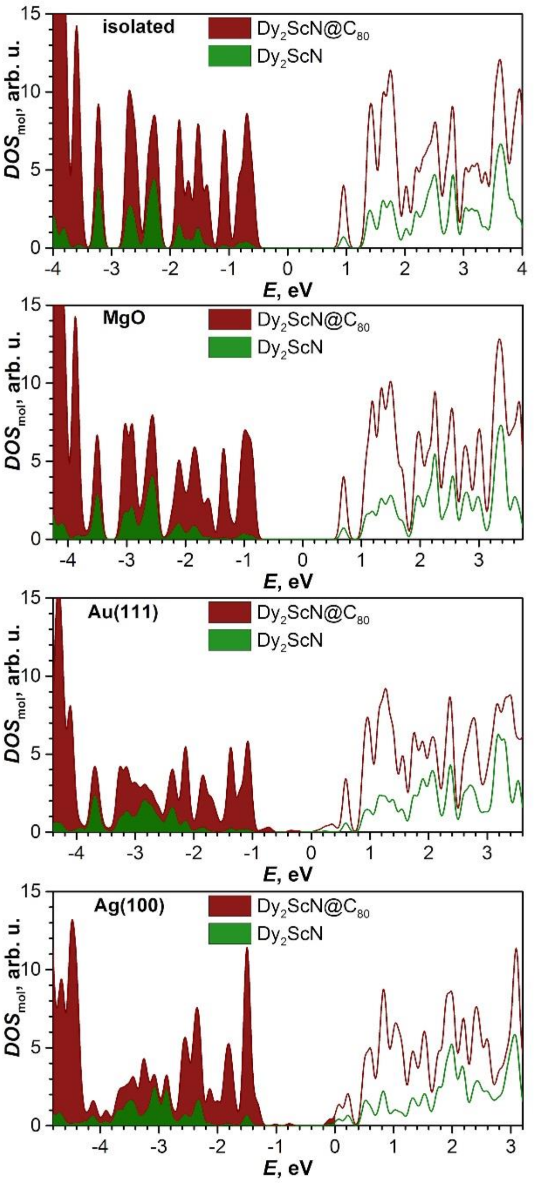

Figure S12. DFT-computed density of states (DOS) near Fermi-level projected onto $Dy_2ScN@C_{80}$ molecule and Dy_2ScN cluster states (dark red and green, respectively; the axis is denoted as DOS_{mol}). The energy scale is shifted from graph to graph to keep HOMO-derived features at approximately the same position.

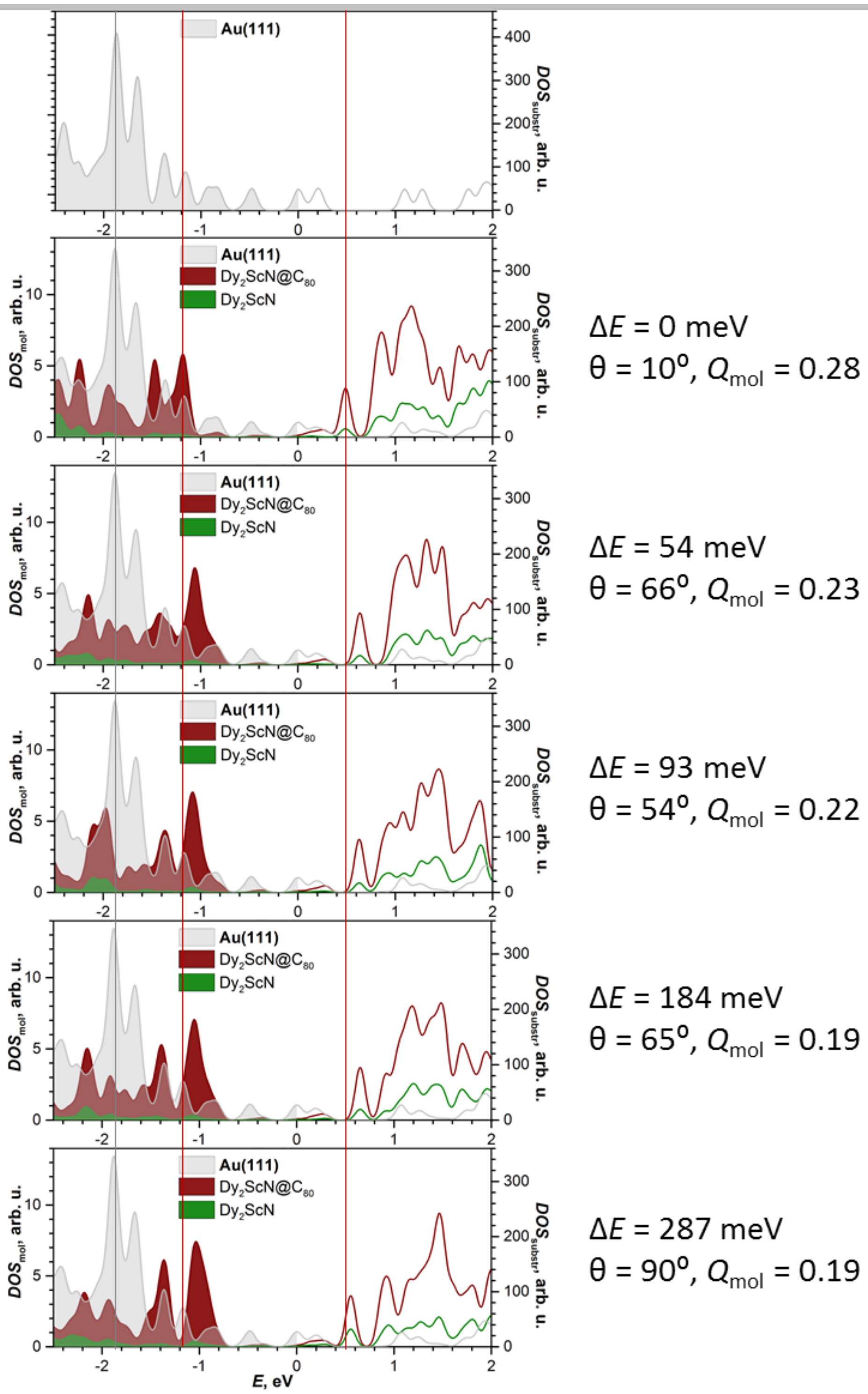

Figure S13. DFT-computed DOS near Fermi-level for five arbitrary chosen conformers of $Dy_2ScN@C_{80}$ on Au(111). Relative energy, cluster tilting angle, and molecular charge are also listed for each conformer. The top panel shows computed DOS of the substrate alone. Vertical lines mark two fullerene and one substrate bands and are added to guide the eye. Note that the major substrate bands are at the same energies, whereas fullerene bands shift from conformer to conformer within the range of 0.2 eV.

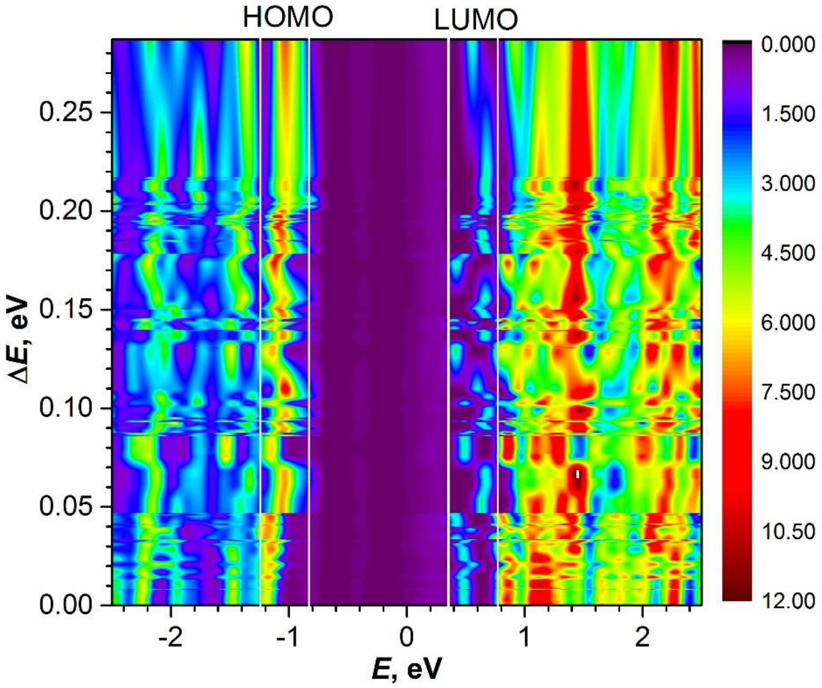

Figure S14. DFT-computed DOS near Fermi-level for all of $Dy_2ScN@C_{80}$ conformers on Au(111) shown as a contour plot. Y axis is the relative energy of the conformer. White lines mark the range of HOMO- and LUMO-derived bands; these bands shift from conformer to conformer within the range of 0.3 eV.

Author Contributions

D.S.K., S.S., and V.D. contributed equally to the work described in the manuscript. D.S.K. performed SQUID magnetometry, STM, and XMCD measurements and was responsible for organization of the beamtime at SLS with help of S.S.; S.S., D.S.K., and T.T.N.N. performed STM measurements in IFW under supervision of C.H. and B.B.; S.S. described the results of these measurements for the manuscript. V.D. performed DFT calculations with help and under supervision of S.M.A.; F.L. and L.S. synthesized $Dy_2ScN@C_{80}$. D.S.K., L.S., C.H.C, C.B., R.W., and A.A.P. performed XAS/XMCD measurements at SLS with help of M.S. and supervision of J.D.; G.V. performed sum-rule analysis with help of R.W.; S.M.A. organized computational studies and performed XAS/XMCD simulations; A.A.P. conceived the study and written the manuscript with contributions from other co-authors.

References

[1] D. S. Krylov, F. Liu, S. M. Avdoshenko, L. Spree, B. Weise, A. Waske, A. U. B. Wolter, B. Büchner, A. A. Popov, *Chem. Commun.* **2017**, *53*, 7901-7904.

[2] a) J. Hafner, *J. Comput. Chem.* **2008**, *29*, 2044-2078; b) G. Kresse, J. Hafner, *Phys. Rev. B* **1993**, *47*, 558-561; c) G. Kresse, D. Joubert, *Phys. Rev. B* **1999**, *59*, 1758-1775; d) J. P. Perdew, K. Burke, M. Ernzerhof, *Phys. Rev. Lett.* **1996**, *77*, 3865-3868; e) S. Grimme, *WIREs Comput. Mol. Sci.* **2011**, *1*, 211-228.

[3] A. Uldry, F. Vernay, B. Delley, *Phys. Rev. B* **2012**, *85*, 125133.

[4] G. Henkelman, A. Arnaldsson, H. Jónsson, *Comput. Mater. Sci.* **2006**, *36*, 354-360.
